# Correlates of Loneliness in Parkinson Disease During the COVID-19 Pandemic: A Longitudinal Study

**DOI:** 10.3390/bs15091233

**Published:** 2025-09-10

**Authors:** John M. de Figueiredo, Robert Kohn, Amar S. Patel, Elijah Parsons, Elan D. Louis, Brian B. Koo

**Affiliations:** 1 Department of Psychiatry, Yale University School of Medicine, New Haven, CT 06510, USA; 2 Department of Epidemiology, Brown University School of Public Health, Providence, RI 02903, USA; robert_kohn@brown.edu; 3 Department of Neurology, Yale University School of Medicine, New Haven, CT 06510, USA; amar.patel@yale.edu (A.S.P.); brian.koo@yale.edu (B.B.K.); 4 The Warren Alpert Medical School, Brown University, Providence, RI 02903, USA; elijah.parsons@alumni.brown.edu; 5 Department of Neurology, University of Texas Southwestern, Dallas, TX 75390, USA; elan.louis@utsouthwestern.edu

**Keywords:** COVID-19, loneliness, Parkinson disease, demoralization, emotional support

## Abstract

Parkinson disease (PD) patients are particularly vulnerable to the effects of loneliness. The objective of this longitudinal study was to assess how the COVID-19 pandemic affected loneliness in PD patients by identifying the correlates of loneliness during the pandemic in the US and to establish a rationale for providing emotional support and restoring morale. Consecutive PD outpatients were recruited during June 2016–May 2017. Data on sociodemographic, clinical, and psychological variables were obtained. During October–December 2020, participants were mailed a questionnaire about some of the variables studied at baseline and new variables specifically related to the pandemic. Univariate, bivariate, and forward linear regression analyses were used to identify the correlates of loneliness. Sex, demoralization, and baseline PD health-related quality of life were significantly associated with loneliness during COVID-19 pandemic, with women reporting more loneliness than men. To examine loneliness specifically associated with the COVID-19 pandemic, loneliness prior to the pandemic was controlled, with only sex and demoralization remaining statistically significant. Interventions aimed at restoring morale and providing emotional support should be included as an essential component of any treatment plan designed to alleviate loneliness during public health emergencies that require social isolation, such as a pandemic.

## 1. Introduction

As social beings, emotional support from companionship is crucial for our good health and well-being, sharing its importance with basic needs, such as food and shelter (Reblin & Uchino, 2008). In particular, loneliness, the distress associated with a perception of isolation, is correlated with a higher risk of morbidity and mortality (Hawkley & Cacioppo, 2010). As we progress into the 21st century, feelings of loneliness and social isolation have become increasingly prevalent (Kannan & Veazie, 2022; Jeste et al., 2023).

In March 2020, the COVID-19 pandemic unexpectedly impacted the United States. The trend of increasing loneliness intensified globally during the pandemic’s quarantine phase and has continued at high rates since then. Noting that nearly half of the adults in the United States report experiencing loneliness prompted the Surgeon General to declare an “epidemic of loneliness” in the country in 2023 (Office of the Surgeon General (OSG), 2023). Similar statements were made by the United States National Academies of Sciences, Engineering, and Medicine et al. (2020) and the World Health Organization (2021). Addressing and understanding the epidemic of loneliness, particularly in the context of public health emergencies that require social distancing, such as the COVID-19 pandemic, stands out as one of the most pressing public health challenges of our time. The social distancing measures necessary to contain the COVID-19 pandemic altered the dynamics between patients and their caregivers. This shift led to behavioral changes that potentially increased caregiver burden, particularly among older patients (Dellafiore et al., 2022).

While loneliness and social isolation are related concepts, they are distinct and may or may not occur together. Social isolation is a diminished quantity and quality of an individual’s social network, whereas loneliness is the distress associated with a discrepancy between desired and perceived social relationships (Peplau et al., 1979). Three major types of loneliness, each reflecting different relational needs, have been identified: “intimate or emotional,” the longing for close, intimate relationships or an emotional partner; “relational or social,” the need for close friendships; and “collective,” the desire for a sense of belonging and community (McDaniels & Subramanian, 2022). A complex biopsychosocial disturbance, loneliness is influenced by genetic heritability (Bowirrat et al., 2023), personality traits (Erevik et al., 2023), socioeconomic status (Bryan et al., 2024), and cultural background (Barreto et al., 2021).

Parkinson disease (PD) is one of the most common neurodegenerative diseases. In 2021, 11.77 million people worldwide had PD. Age-standardized rates of incidence, prevalence, and disability adjusted life years worldwide were 15.63/100,000, 138.63/100,000, and 89.59/100,000, respectively (Luo et al., 2025). PD is a complex disease characterized by heterogeneous presentations of both motor and non-motor symptoms. Although classified as a movement disorder due to motor symptoms, such as tremors, bradykinesia, and rigidity, non-motor symptoms of PD are pervasive and often more disabling than motor symptoms, and include changes in mood, behavior, cognition, sleep, and autonomic functions, such as bowel and bladder control (Bloem et al., 2021). A significant number of patients with PD experience depression, anxiety, apathy, and cognitive deficits (Chaudhuri et al., 2006). Additionally, recent studies indicate that demoralization is common in PD and can be differentiated from depression (Koo et al., 2018; Elfil et al., 2020; Zhu et al., 2021; de Figueiredo et al., 2022; de Figueiredo et al., 2023).

The objective of this longitudinal study was to identify the correlates of loneliness in PD patients during the COVID-19 pandemic in the United States. This study aimed to expand our knowledge of the mental health of PD patients during the COVID-19 pandemic by assessing the added burden imposed by the social restrictions required during the pandemic to a population already vulnerable to biopsychosocial impairments.

## 2. Materials and Methods

### 2.1. Data Collection Procedures

#### 2.1.1. Baseline Study Procedure

The COVID-19 pandemic provided an opportunity to study the impact of loneliness in a sample of PD patients who had participated in an earlier study where several variables could be examined longitudinally. Consecutive PD outpatients from the Movement Disorders Clinic at Yale-New Haven Hospital, a private healthcare system, were recruited from June 2016 to May 2017. Data on the variables listed in Table 1 were collected partly by self-report and partly by trained research assistants following a clinic appointment, after obtaining written informed consent.

#### 2.1.2. COVID-19 Follow-Up Procedures

During October to December 2020, participants in the baseline study received a telephone call from the Principal Investigator (J.M. de F.) requesting their participation in a research study that assessed how they were dealing with the COVID-19 pandemic. They were told that they were selected because they had participated in the baseline study. They were asked if they were willing to receive a questionnaire in the mail to complete and return using an enclosed pre-posted envelope. They were told that the study would involve obtaining information from their medical record and asked not to write their names on the paper forms. Each questionnaire had a cover letter explaining the study and stating that the study is voluntary and that not participating will have no impact on the relationship or care they receive from their providers. A week later, a member of the research staff called the participants, reviewed the letter over the telephone, and documented their agreement to participate in the study in the research record. A follow-up telephone call was made to those individuals whose questionnaires were not returned after three weeks. Refusals and non-responders were tracked to be able to examine response bias. The questionnaires were collected from 10 October 2020 through 9 March 2021. 

#### 2.1.3. Admission and Exclusion Criteria

Admission criteria were: (1) Age between 40 and 90 years; (2) diagnosis of idiopathic PD for more than one month prior to enrollment in this study, with the diagnosis made by a movement disorders neurologist (A.S.P.) according to UK Brain Bank Society criteria (Gibb & Lees, 1988); and (3) English comprehension/literacy. Exclusion criteria were (1) Diagnosis of a major neurocognitive disorder; (2) history of dementia; (3) presence of psychotic symptoms; (4) current alcohol or other substance use or dependence; (5) being deemed legally incompetent; and (6) being near the end of life as defined by hospice admission. Age 40 was the lower limit because younger patients may experience the disease differently due to their unique life circumstances (Schrag et al., 2003). The neurologist (A.S.P.) assessed the presence of psychotic symptoms. Trained research assistants asked a standard set of questions to determine if potential participants were currently using substances or had ever attempted or contemplated suicide, and they validated the replies by reviewing medical records. Current substance users were excluded as use of certain substances may cause Parkinson-like symptoms, complicating the diagnosis and interpretation of the findings (Vasconcelos et al., 2022).

### 2.2. Assessments

#### 2.2.1. Variables and Measurements

Table 1 provides an overview of the variables and instruments used during the baseline and the COVID-19 follow-up. The dependent variable was loneliness during the COVID-19 follow-up.

The independent variables included in the baseline study were sociodemographic variables; cognition; variables related to PD (number of years since PD diagnosis, dyskinesia, disease stage); health history and treatment; anxiety; depression; subjective incompetence; demoralization; perceived stress; perceived social support; resilience; PD healthcare related quality of life (PDHRQoL); and activities of daily living. We included questions on current or past cigarette smoking and alcohol use.

The independent variables assessed in the COVID-19 follow-up were sociodemographic variables; general health perception; variables related to health history, including COVID medical risk; anxiety; depression; subjective incompetence; demoralization; perceived stress; perceived emotional–informational social support; resilience; PDHRQoL; family functioning; and variables relevant to the pandemic. Included were questions on cigarette smoking, alcohol use, and cannabis use (current or past and change, i.e., increase, decrease, or no change) since the COVID-19 pandemic. Pre-COVID function was assessed during the follow-up study regarding perceived emotional–informational social support, family functioning, and loneliness.

The survey included questions on the impact of the pandemic, comorbid physical conditions placing the subjects at high risk (lung disease/asthma, heart disease, diabetes, liver disease, obesity, dialysis, immunocompromised); testing for COVID-19; if tested, was it positive for COVID-19; getting ill from COVID-19; living with someone who has COVID-19; or knowing someone who became ill from COVID-19; knowing someone who died from COVID-19; how much the pandemic impacted day-to-day life; and adherence to COVID-19 prevention recommendations. In addition, COVID-19 scales were included that measured fear, anxiety, obsession, and post-traumatic stress disorder (PTSD) due to COVID-19.

#### 2.2.2. Description of Scales

##### Loneliness (LON)

Loneliness, the dependent variable, was assessed during the COVID-19 follow-up using the three-item version of the Revised UCLA Loneliness Scale (R-UCLA) (LON) (Hughes et al., 2004). LON scale was asked twice: once about current loneliness and again retrospectively about loneliness prior to COVID-19. This is a shortened version of the original 20-item scale (R-UCLA) designed to measure one’s subjective feelings of loneliness as well as feelings of social isolation (Russell et al., 1980). The scale focuses on three key dimensions of loneliness: relational connectedness, social connectedness, and perceived isolation, with the following questions: “How often do you feel that you lack companionship? How often do you feel left out? How often do you feel isolated from others?” Participants rate each item as 1 (hardly ever); 2 (some of the time); or 3 (often). As in the R-UCLA scale, each person’s responses to the questions are summed, with higher scores indicating greater loneliness and a possible range of 3 to 9. There is no universally recognized cutoff point and loneliness was assumed to be continuous. The three-item version of the R-UCLA has been studied in investigating PD and loneliness (Prell et al., 2023), as well as investigations of COVID-19 (Benke et al., 2022; Liu et al., 2024).

##### General Health Perceptions (SF-36)

The SF-36 (36-item Short Form survey) General Health Perceptions Subscale was used to evaluate perceived health and well-being (Ware & Sherbourne, 1992). It is one of the eight self-report subscales within the 1992 SF-36 questionnaire. It consists of five questions that assess aspects like feeling that health is improving, declining, or staying the same, and how it affects daily life.

##### COVID-19 Medical Risk

COVID-19 medical risk was assessed by asking yes/no questions if the participant had one or more of the following: lung disease/asthma, heart disease, diabetes, liver disease, obesity, dialysis, or immunocompromised.

##### Cognition (GPCOG)

Trained research assistants administered the General Practitioner Assessment of Cognition (GPCOG) Screening Test (Brodaty et al., 2002). This test consists of giving first a name and address for subsequent recall, then asking questions about the date (1 item), clock drawing (2 items), information on current events (1 item), and recall of the name and address previously given (5 items). The score ranges from 0 to 9, with 0 to 4 indicating cognitive impairment, 5 to 8 requiring additional information from an informant, and 9 meaning no cognitive impairment.

##### Dyskinesia and Motor Function (MDS-UPDRS-M)

The neurologist evaluated the participants for dyskinesia and motor function using Part III of the Movement Disorder Society Sponsored Revision of the Unified Parkinson’s Disease Rating Scale for motor symptoms (MDS-UPDRS-m) (Postuma et al., 2015). The MDS-UPDRS-m Part III assesses problems in motor function on a 5-point scale: 0 (no problems), 1 (minimal), 2 (mild), 3 (moderate), and 4 (severe).

##### PD Stage (H-Y)

The neurologist evaluated and classified PD stage using the Hoehn and Yahr (1967) staging classification (H-Y). Scored from the history given by patients and caregivers, and a medical examination. H-Y distinguishes five severity stages based on the extent of involvement and functional impairment. 1) Unilateral involvement usually with minimal or no functional disability; (2) Bilateral or midline involvement without impairment of balance; (3) Bilateral disease with mild to moderate disability and impaired postural reflexes but physical independence; (4) Severely disabling disease but still able to walk or stand unassisted; and (5) Confinement to bed or wheelchair unless aided.

##### PDHRQoL (PDQ-8)

The neurologist assessed PDHRQoL using the Parkinson Disease Questionnaire-Short Form (PDQ-8) (Jenkinson et al., 1997a), a shortened version of the PDQ-39 (Jenkinson et al., 1997b), a more comprehensive measure. The PDQ-8 has one question selected from each of the eight domains of PDQ-39 (mobility, activities of daily living, emotional well-being, stigma, social support, cognitions, communication, and bodily discomfort). Each question is scored from 0 to 4, and the scores are summed and transformed to a percentage score between 0 and 100. PDQ-8 provides a single index score representing overall PDHRQoL.

##### Anxiety (GAD-7)

Anxiety was assessed with the Generalized Anxiety Disorder-7 (GAD-7) (Spitzer et al., 2006). This is a self-report scale with 7 items based on the DSM-IV criteria for generalized anxiety disorder. Scores on each item range from “0” (“not at all”) to “3” (nearly every day). The scale measures the frequency of symptoms of anxiety over the past two weeks. The total score ranges from 0 to 21, with higher scores indicating more severe anxiety. A cut-off of ≥10 is frequently used to suggest a potential clinical case of anxiety. The GAD-7 has been used in COVID-19 loneliness studies (Liu et al., 2024).

##### Depression (PHQ-9)

Depression was assessed with the Patient Health Questionnaire (PHQ-9) (Kroenke et al., 2001). This is a self-report scale with 9 items based on the DSM-IV criteria for major depressive disorder. Scores on each item range from “0” (“not at all”) to “3” (nearly every day). The scale measures the frequency of symptoms of depression over the past two weeks. The total score ranges from 0 to 27, with higher scores indicating more severe depression. A cut-off of ≥10 is frequently used to suggest a potential clinical case of depression. Depression using the PHQ-9 has been examined in research of the association of loneliness and COVID-19 (Liu et al., 2024).

##### Subjective Incompetence (SIS)

Proposed as the clinical hallmark of demoralization, subjective incompetence is “a self-perceived incapacity to perform tasks and express feelings deemed appropriate in a situation perceived as stressful, resulting in pervasive uncertainty and doubts about the future” (de Figueiredo & Frank, 1982). Subjective incompetence occurs when people face a stressor that contradicts their assumptions about themselves and others and may progress to feelings of helplessness and hopelessness. Individuals with subjective incompetence are puzzled, indecisive, uncertain, and unclear about how to handle a stressful situation, leaving them in a quandary. The Subjective Incompetence Scale (SIS) (Cockram et al., 2009) is a 12-item, self-report scale, scored on a 4-point Likert scale from “none of time” to “most of time,” assessing subjective incompetence in the past two weeks with such items as “being puzzled, indecisive, and uncertain as to what actions, if any, should be taken” or “running out of ideas on how to handle the situation.” Three items are reverse scored. In PD patients, subjective incompetence is recognized as a key factor contributing to demoralization, independent of depression and anxiety (de Figueiredo et al., 2022). The relationship of loneliness and subjective incompetence has not previously been studied.

##### Demoralization (DS and DS-II)

The Demoralization Scale (DS) (Kissane et al., 2004) used in the baseline study has 24 items, rated on a 5-point Likert scale from 0 (never) to 4 (all the time), with a range from 0 to 96. The total score is the sum of the scores on 5 subscales (loss of meaning and purpose, dysphoria, disheartenment, helplessness, and sense of failure), with higher scores indicating higher demoralization. The Demoralization Scale-II (DS-II) is a 16-item scale derived from the DS (Robinson et al., 2016b), rated on a 3-point Likert scale, including 0 (never), 1 (sometimes), and 2 (often), with a range from 0 to 32 and higher scores indicative of higher levels of demoralization (score range, 0–32). The scale contains two 8-item factors, “meaning and purpose” and “distress and coping ability.” Both scales are self-report and based on the assumption that demoralization is continuous. To be able to compare DS-II to baseline demoralization a variable containing only the 16 items found in DS-II was created from DS, and both scales were converted to z-scores. A link between demoralization and loneliness has been hypothesized; however, studies linking the two constructs are lacking (Vehling & Kissane, 2018).

##### Perceived Stress (IES)

Perceived stress was measured with the Impact of Event Scale (IES) (Horowitz et al., 1979). The IES is a 15-item self-report scale in which being bothered by a series of difficulties during the past 7 days is rated from “not at all” to “extremely.” The scale was designed to measure the distress experienced after a specific stressful life event and has been used as a screen for probable post-traumatic stress disorder (PTSD).

##### Perceived Social Support (ISEL-SF and BS-6-SC)

Perceived social support was assessed using a short form of the Interpersonal Support Evaluation List (ISEL-SF). The ISEL is a self-report scale used to assess the perceived availability of social support. The original scale has 40 items, but a shortened 16-item scale was used in the baseline procedure. Each is a statement to be rated on a 4-point scale ranging from “definitely false” to “definitely true” (Sanderson, 2019).

The Brief Social Support Scale (BS-6-SC) (Beutel et al., 2017) is a 6-item self-report scale derived from the longer 19-item Medical Outcomes Study Social Support Survey (MOS-SSS) (Sherbourne & Stewart, 1991). The 3-item emotional–informational social support subscale of the BS-6-SC assesses the perceived adequacy of support from family, friends, and significant others in terms of emotional and informational needs, including items related to receiving support, comfort, advice, and guidance from others. The ISELF and BS-6-SC have not been used in loneliness or COVID-19 studies previously. Multiple studies have been conducted during COVID-19 demonstrating the association between social support and loneliness using various social support instruments (Gabarrell-Pascuet et al., 2023).

##### Resilience (BRS)

The Brief Resilience Scale (BRS) (Smith et al., 2008) is a self-report scale with 6 questions (3 positively and 3 negatively worded). It is scored on a 5-point Likert scale from “strongly agree” to “strongly disagree.” The BRS was used in a COVID-19 study to show that resilience decreased loneliness (Pineda et al., 2022).

##### Family Functioning (BAFFS)

The Brief Assessment of Family Functioning Scale (BAFFS) (Mansfield et al., 2019) is a three-item scale scored on a 4-point Likert scale from “strongly agree” to “strongly disagree.” The higher the score, the worse the family functioning. Poorer family functioning using the Family Assessment Device (Mansfield et al., 2015), from which the BAFFS was developed, has been associated with loneliness (G. Wang et al., 2011).

##### Activities of Daily Living (ADLs)

The presence or absence of disability in activities of daily living was assessed for bathing, dressing, getting out of a chair, and walking (Gill et al., 2002). The numbers of activities of daily living resulting from the presence of disability were summed.

##### Fear of COVID-19 (FC-19S)

The Fear of COVID-19 Scale (FC-19S) (Ahorsu et al., 2020) is a 7-item self-report scale that assesses the severity of COVID-19 fear with statements such as “I am Coronavirus-19”. Each item is rated on a 5-point Likert scale ranging from 1 (“strongly agree”) to 5 (“strongly disagree”) with scores ranging from 7 to 35 and higher scores indicating greater fear of COVID-19.

##### Coronavirus Anxiety (CAS)

The Coronavirus Anxiety Scale (CAS) (Lee, 2020a; Lee et al., 2020) is a 7-item self-report scale designed to identify probable cases of dysfunctional anxiety associated with the COVID-19 crisis. Each item refers to a symptom of anxiety in the past two weeks, rated for frequency ranging from 0 (not at all) to 4 (nearly every day).

##### Obsession with COVID-19 (OCS)

The Obsession with COVID-19 Scale (OCS) (Lee, 2020b) is a 4-item self-report scale designed to assess the frequency of persistent and disturbed thinking about the COVID-19 pandemic. Each item of the OCS is rated on a 5-point Likert scale, ranging from 0 (not at all) to 4 (nearly every day), based on experiences over the past two weeks.

##### Exposure to Trauma and PTSD (BTQ and PC-PTSD-5)

The Brief Trauma Questionnaire (BTQ) (Schnurr et al., 1999) is a self-report sale that asks if the respondent was ever exposed to 10 traumatic events. If exposure occurred, the respondent was asked to complete the Primary Care PTSD-5 Scale to evaluate for PTSD unrelated to COVID-19. The Primary Care PTSD-5 Scale (PC-PTSD-5) (Prins et al., 2016) is a self-report screen designed to identify probable PTSD in primary care settings. The scale begins by asking if the respondent was ever exposed to a traumatic event. If exposure is denied, the screen is complete with a score of 0. If exposure is admitted, five additional yes/no questions inquire how that trauma exposure has affected the respondent over the past month. Answering at least three of the five items positively was presumptive of PTSD. The PC-PTSD-5 was also used to evaluate PTSD due to COVID-19 by linking the items to the pandemic.

### 2.3. Statistical Analysis

#### 2.3.1. Bias Analysis

A bias analysis was conducted comparing participants and non-participants of the follow-up study using independent *t*-tests and chi-square tests.

#### 2.3.2. Bivariate Analyses

Statistical differences in measures between baseline and the COVID-19 follow-up were conducted using the McNemar test for paired nominal data and paired *t*-tests for means. Bivariate associations with loneliness were studied with Pearson correlations for continuous variables and *t*-tests for categorical variables (Siegel & Castellan, 1988). Table 2 provides the Cronbach alpha for the scales and their *t*-test reliability.

#### 2.3.3. Regression Analyses

Linear forward regressions were conducted using loneliness during the follow-up period as the dependent variable (Rencher & Schaalje, 2008) and using those variables that were statistically significant (*p* < 0.05) in the bivariate analysis as independent variables. The first regression used only those variables that were assessed during the COVID-19 follow-up. The second regression used only those variables that were assessed during the baseline study. The third regression combined those independent variables that produced the best-fitting model in the first and second sets of forward regressions. This model produced the best correlates for loneliness during COVID-19. To determine the best correlates specifically associated with the COVID-19 pandemic, a fourth regression was conducted, controlled for loneliness during the pre-COVID-19 period, using the results of the third model.

## 3. Results

At baseline, a total of 133 patients who met the admission criteria were invited to participate. Of these, 95 agreed to participate in the baseline assessment and 38 declined, giving a participation rate of 71.4%. Participants and non-participants were compared for sex, age, marital status, and H-Y stage. The 38 who declined had similar sex, age, and marital status to the 95 who agreed to participate, but they were more likely to have more severe PD, i.e., to be classified as belonging to H-Y stage III or IV (39.5% vs. 11.5%, *p* < 0.0002).

Of the 95 subjects who had participated at baseline, 55 (57.9%) participated in the follow-up study. Among those who did not participate in the follow-up, at least 6 were not available to participate because they were deceased.

### 3.1. Bias Analysis Results

A comparison of those who participated and did not participate in the follow-up study found no significant differences in the distributions by sex, age, marital status, race-ethnicity, number of individuals in the household, education, and the following variables assessed at baseline: cognition, resilience, anxiety, demoralization, perceived stress, perceived social support, and number of years since diagnosis. There were no statistically significant differences in dyskinesia or movement disorder as measured by MDS-UPDRS-m scores. However, differences were found with other measures of PD, with those who did not participate having more severe disease, as portrayed by PDQ-8 (t = 2.5, df = 93, *p* < 0.02) and H-Y stage (t = 2.8, df = 91, *p* < 0.006). Additionally, subjective incompetence (t = 2.8, df = 93, *p* < 0.007) and depression (PHQ-9 ≥ 10) (χ^2^ 8.1, df = 1, *p* < 0.004) were greater among non-participants.

### 3.2. COVID-19 Follow-Up Univariate Analysis

The majority of the respondents were men (61.8%), married (72.7%), white (92.7%), and college educated (76.4%). The average age of the respondents was 70.8 ± 8.8. Less than half of the respondents were tested for COVID-19 (41.8%); only two became ill from the virus. A third (32.7%) of their family members had COVID-19 but only two individuals had someone they lived with who became ill. The mean score of loneliness during the COVID-19 pandemic was 1.9 ± 1.8.

### 3.3. Paired Analysis of Baseline and COVID-19 Follow-Up

Table 3 shows the results of the paired analyses of the variables evaluated both at baseline and follow-up, as well as the retrospective pre-COVID-19 recall for LON, BS6, and BAFFS. LON was significantly higher during the COVID-19 pandemic compared to pre-COVID-19 (*p* < 0.04). Household size was smaller during the follow-up compared to baseline (*p* < 0.001). PDHRQoL had worsened during follow-up (*p* < 0.003). Subjective incompetence was greater at follow-up (*p* < 0.001), and resilience was lower compared to baseline (*p* < 0.001).

### 3.4. Bivariate Analysis

Table 4 presents the Pearson correlations of continuous variables with loneliness during follow-up. Among the variables assessed at baseline, increased subjective incompetence and increased demoralization were significantly correlated with increased loneliness. Reduced PDHRQoL, less resilience, and lower social support were correlated with increased loneliness. PD severity baseline measures (MDS-UPDRS and H-Y) were not associated with loneliness. Lower emotional–informational social support, poorer family functioning, and loneliness based on retrospective recall were associated with greater loneliness during follow-up.

Variables with statistically significant correlations with increased COVID-19 loneliness at follow-up included poorer family functioning and lower emotional–informational social support. Having greater demoralization at follow-up, more subjective incompetence and less resilience were significantly correlated with increased loneliness. Reduced PDHRQoL significantly correlated with increased COVID-19 loneliness. COVID-19 related variables, including household size, impact of the COVID-19 pandemic on one’s life, COVID-19 fear, COVID-19 anxiety, and COVID-19 obsession, were not associated with loneliness.

Only a few categorical variables were associated with loneliness during the COVID-19 pandemic (see Table 5). Women were significantly more likely to feel lonely than men. Other statistically significant associations with loneliness during the COVID-19 pandemic were not being married during the pandemic and having depression at follow-up. Only one respondent had PTSD pre-COVID-19 and two had PTSD due to the COVID-19 pandemic.

### 3.5. Multivariate Analyses

Forward linear regressions were conducted using loneliness during follow-up as the dependent variable and variables that had statistically significant associations with the dependent variable in bivariate analyses as independent variables (see Table 6). In the forward regression to determine the best fitting risk factors of loneliness during the COVID-19 pandemic based on follow-up variables only (Model 1) sex, demoralization, and perceived emotional–informational social support remained statistically significant in the model. Depression, family functioning, had COVID-19, marital status PDHRQoL, resilience, sex, subjective incompetence during COVID-19 follow-up did not remain in Model 1. There was no significant interaction between demoralization and sex. Collinearity was not an issue among the variables in Model 1, and a normal Q-Q plot of the standardized residuals suggested that the data were normally distributed (Shapiro Wilk = 0.98, df = 53, *p* < 0.54).

Only, PDHRQoL remained statistically significant as an independent variable in the forward regression examining loneliness during the COVID-19 pandemic and baseline variables (Model 2). Family functioning and perceived emotional–informational social support prior to COVID-19 did remain in Model 2; as well as demoralization, social support and subjective incompetence at baseline. A normal Q-Q plot of the standardized residuals suggested that the data in Model 2 were normally distributed (Shapiro Wilk = 0.97, df = 55, *p* < 0.11).

When the baseline and follow-up variables from models 1 and 2 were combined (Model 3) only sex, demoralization during the COVID-19 pandemic, and PDHRQoL at baseline remained statistically significant, and perceived emotional–informational social support during the COVID-19 pandemic showed a trend (*p* < 0.06). The results of Model 3 showed the best model for risk factors associated with loneliness in PD during the COVID-19 pandemic. Collinearity was not an issue among the variables in Model 3, and a normal Q-Q plot of the standardized residuals suggested that the data were normally distributed (Shapiro Wilk = 0.97, df = 53, *p* < 0.31).

In Model 4 the independent variables in Model 3 were controlled for loneliness prior to the COVID-19 pandemic to determine what were the best correlates of loneliness in PD specifically associated with the COVID-19 pandemic, and only sex and demoralization during the COVID-19 pandemic remained significant. Collinearity was not an issue among the variables in Model 4, and a normal Q-Q plot of the standardized residuals suggested that the data were not normally distributed (Shapiro Wilk = 0.91, df = 51, *p* < 0.001).

F test for nested models was used to compare the various models. Improvements in adjusted R^2^ and AIC (Akaike Information Criterion) were as follows: From Model 1 to Model 3, adjusted R^2^ improved by 0.31 and AIC by 7 (meaningful evidence in favor of Model 3) with Model 3 providing a significantly better fit to the data (F = 10,466; df = 1, 48; *p* < 0.003); from Model 2 to Model 3, adjusted R^2^ improved by 0.5 and AIC by 19 (strong evidence in favor of Model 3), with Model 3 providing a significantly better fit to the data (F = 9278; df = 3, 48; *p* < 0.0006); from Model 3 to Model 4, adjusted R^2^ improved by 0.05 and AIC by 34.9 (strong evidence in favor of Model 4), with Model 4 providing a significantly better fit to the data (F = 41,748; df = 1, 45; *p* < 0.0001).

## 4. Discussion

This study of outpatients with PD expands our knowledge of loneliness during the COVID-19 pandemic by measuring variables not previously assessed, such as demoralization, subjective incompetence, family functioning, emotional social support, and variables specifically related to the pandemic. Participants from a cross-sectional study conducted before the COVID-19 pandemic were re-evaluated during the pandemic through a mail survey. The assessments included variables previously studied at baseline and new variables not evaluated before, including variables specifically relevant to the pandemic. After conducting a series of regression analyses to determine the best correlates of loneliness during the pandemic, only sex (with women experiencing more loneliness than men) and greater demoralization during the pandemic and poorer PDHRQoL at baseline remained statistically significant. When loneliness specifically associated with the pandemic was examined by controlling for loneliness before the pandemic, only sex and demoralization during the pandemic remained statistically significant.

A scoping review of 31 studies that examined the impact of the COVID-19 pandemic on PD patients identified six major themes: COVID-19 concerns, access to healthcare, and the impact of the pandemic on physical and mental health, daily and social activities, physical activity, and caregivers (Brooks et al., 2021). Mental health impacts were noted in many studies to be worse for women, those with longer disease duration, pre-existing mental health problems, reduced physical activity, and reduced disease severity. The review concluded that “the COVID-19 pandemic has had negative effects on the physical and mental health of people with PD, perhaps due to disruption of healthcare services, loss of usual activities and supports, and reduction in physical activity” (Brooks et al., 2021). These areas of study are echoed broadly by other meta-reviews. Several studies documented an increase in anxiety, depression, and sleep problems (Nabizadeh et al., 2022); a significant increase in depressive symptoms when compared to healthy controls or other neurologic conditions (Mameli et al., 2022); a worsening of both motor and non-motor symptoms during six months (Shalash et al., 2022); an increase in depression, sleep problems, rigidity, and tremors; and an increase in anxiety and stress due to lack of access to treatment and decreased physical activity (Afraie et al., 2023). However, PD patients did not have a higher risk of contracting COVID-19 or being hospitalized or dying from COVID-19 (Afraie et al., 2023).

Studies comparing the prevalence of loneliness in patients with PD to healthy controls have produced conflicting results. One study reported a high prevalence of loneliness ranging from 25% to 53%, depending on the cutoff point used (Prell et al., 2023), while another study found no statistically significant difference between the two groups (Shahmoon et al., 2025). Although patients with PD are at risk for loneliness, it remains unclear whether they are more vulnerable to it compared to healthy controls or other groups with different health conditions. Nevertheless, individuals who report being lonelier are significantly more likely to develop PD. In participants of a large cohort study, followed for up to 15 years, loneliness was significantly associated with an increased risk of incident PD after controlling for demographic and socioeconomic factors, social isolation, genetic risk, and physical and mental health (Terracciano et al., 2023).

PD patients are particularly vulnerable to the effects of loneliness. This is partly because loneliness has been linked to the onset of neurodegenerative diseases (Salinas et al., 2022), and partly because PD patients are typically over the age of 60, a group often experiencing loneliness due to reduced social interaction and declining health (Gerlach et al., 2024). Furthermore, loneliness is a significant biopsychosocial stressor in adults with chronic physical illnesses overall (Petitte et al., 2015).

PD patients are at risk of social isolation and loneliness due to the prevalence of non-motor symptoms and symptoms that disrupt social functioning, such as difficulty expressing emotions (facial masking), problems modulating vocal rhythms, and difficulties recognizing emotions in others’ faces and voices. These socially relevant symptoms can create challenges for people with PD in maintaining their interpersonal relationships (Prenger et al., 2020). Additionally, PD patients often experience stigma correlated with the severity of their symptoms, whether perceived by the patient or openly acknowledged by caregivers or bystanders. Perceived stigma and the severity of non-motor symptoms are the strongest predictors of loneliness among these patients (Shahmoon et al., 2025). To sum up, loneliness is a major risk factor for the appearance of new cases of PD and complicates the course of PD by creating a vicious circle in which the symptoms of the illness exacerbate the loneliness and vice versa.

Studies that have explored the impact of loneliness on the symptom burden of PD during the COVID-19 pandemic suggest a strong reciprocal relationship. In a cross-sectional study where the diagnosis of idiopathic PD was self-reported by the participants, increased feelings of loneliness were correlated with a greater severity of both motor and non-motor symptoms and lower health-related quality of life (HRQoL) (Subramanian et al., 2020). A longitudinal study of 80 participants with early PD compared social isolation, symptoms of PD, and HRQoL before, during, and after the COVID-19-related social restrictions. This study did not measure loneliness directly but found an association of social isolation due to COVID-19 restrictions with worsening of both motor and non-motor symptoms of PD and of HRQoL (Mehta et al., 2024). The bivariate analysis showed that PDHRQoL had decreased during the follow-up suggesting that PD had progressed among the patients.

Loneliness has other biological impacts beyond PD symptomatology. Research has found an association between loneliness, stress-related inflammatory, and neuroendocrine responses, as well as with increased hypothalamic–pituitary–adrenal axis activity (Steptoe et al., 2013). Studies have demonstrated neuroimaging changes in brain structure and functioning across a number of neurobiological assessments (Lam & Lee, 2023).

The results of our study align with previous reports indicating that women were more susceptible to loneliness during the pandemic than men among those aged 60 or older (Wickens et al., 2021). A new finding is that demoralization, not examined in previous studies, had a strong association with loneliness whereas depression and anxiety failed to reach statistical significance when demoralization was included in the regression models. These results underscore the importance of diagnosing and addressing demoralization related to loneliness and social isolation, particularly among women. Interestingly, none of the pandemic-specific variables were associated with loneliness. This may be due to social isolation already being associated with PD regardless of the pandemic as well as the use of longstanding arrangements already in place to mitigate and manage the social deprivation associated with the COVID-19 pandemic (Bundy et al., 2021; Heimrich et al., 2023; Styslinger et al., 2023).

Demoralization, a treatable condition, is characterized by symptoms of distress, such as inability to cope, sense of failure, and loss of purpose and meaning, together with a feeling of entrapment (subjective incompetence) that can sometimes progress to helplessness, hopelessness, existential despair, demands for hastened death, and a desire for suicide or death (Frank et al., 2025; de Figueiredo & Frank, 1982; Tecuta et al., 2015; Robinson et al., 2015; Robinson et al., 2016a).

Understanding the difference between depression and demoralization is essential for effective treatment planning, especially in patients with PD. Depression and demoralization have distinct presentations and follow different trajectories, necessitating tailored interventions for each. While they can co-occur, their overlap is relatively limited. In cases of depression, individuals often experience anhedonia (a loss of pleasure) and anergia (a lack of energy), and have a reduced magnitude of motivation to deal with their stressful situation, even when they are aware of the actions they should take. Demoralization, on the other hand, does not involve anhedonia or anergia. Instead, individuals who are demoralized face difficulties in overcoming their challenges due to uncertainty regarding the appropriate actions to take (direction of motivation). This uncertainty is “subjective incompetence” and may be viewed as a manifestation of an absence of a cognitive map to deal with the stressful situation (de Figueiredo, 1993).

Our earlier studies found that demoralization in PD has a prevalence rate of 18.1% (Koo et al., 2018); patients with PD are more likely than control subjects to have lifetime histories of both depression and demoralization (Elfil et al., 2020); demoralization better accounts for disruptions in quality of life compared to depression (Zhu et al., 2021); and resilience appears to be a mechanism evolved to protect against demoralization, not just depression (de Figueiredo et al., 2023). Furthermore, suicide rates are higher among PD patients compared to the general population, even when accounting for other mental disorders (Chen et al., 2021).

Therapeutic interventions have been proposed to address loneliness in later life. Van Orden and Conwell have classified these interventions based on the pathways that contribute to the development of loneliness. Examples include: (1) psychotherapy, reminiscence therapy, mindfulness, and groups to address mental health disorders and psychological needs; (2) health promotion classes and social prescribing to tackle physical health issues; (3) technology platforms, home-delivered meals, and robotic and real pets to assist with functional impairments; and (4) care management, volunteering, senior centers, and opportunities for companionship to reduce social stressors. They also emphasize the importance of considering cultural context, including societal prejudices such as racism and ageism, as well as access to healthcare resources (Van Orden & Conwell, 2023).

Therapeutic interventions are also available for demoralization (Griffith & Gaby, 2005; Kissane, 2017; Y. Wang et al., 2023; Dong et al., 2025). One key aspect of demoralization is subjective incompetence that can be alleviated by altering how individuals perceive stress, restoring hope, and replacing negative thought patterns about themselves and challenging situations with more positive, accurate, and realistic perspectives. Cognitive-behavioral psychotherapy (Beck et al., 2024), for instance, has been effective in countering demoralization, especially when combined with other approaches, such as meaning-centered psychotherapy (Rodin et al., 2018; Kissane et al., 2019) and well-being therapy (Fava, 2016). Well-being therapy enhances resilience, which is the opposite of subjective incompetence, by guiding the patient towards an optimal level in the six dimensions of psychological well-being (environmental mastery, personal growth, purpose in life, autonomy, self-acceptance, and positive relations with others) (Fava, 2016). A sequential combination of cognitive-behavioral therapy and well-being therapy has shown success in treating depressed patients with acute coronary syndromes (Rafanelli et al., 2020). The clinical utility of these therapeutic methods and strategies should be evaluated in PD. Furthermore, interpersonal psychotherapy has been shown to effectively address anxiety, depression, and PTSD (Markowitz, 2021). Given that demoralization is a risk factor for PTSD (Kohn, 2013), additional research is necessary to investigate the potential benefits of interpersonal psychotherapy in this context.

The findings suggest that a multifaceted approach to improve and maximize PDHRQoL, including medication management, lifestyle adjustments, physical therapy, speech therapy, occupational therapy, and psychotherapy would reduce the risk of loneliness during a future public health emergency requiring social isolation (Hwang et al., 2020; Zhao et al., 2021).

The findings highlight the need for further research to assess the effectiveness of integrating interventions aimed at addressing demoralization with those designed to reduce loneliness, such as socially engaged psychotherapy (Van Orden et al., 2021), in patients with PD. To our knowledge, this type of evaluation has not yet been conducted. This synthesized research could be especially valuable during public health emergencies, like a pandemic, when isolating patients is necessary. Since multiple pathways can lead to feelings of loneliness and demoralization, treatment interventions should be personalized to meet individual needs. As noted by Costanza et al. (2020), “it is vital to explore the various components of demoralization and the meanings that patients attribute to them.” They correctly state that “this exploration is important because it helps reshape patients’ perspectives and ultimately reinforces their sense of purpose, which may alleviate their suffering.” Additionally, as highlighted by Van Orden and Conwell (2023), it is essential to identify the specific pathways that contribute to loneliness and to select appropriate interventions based on those insights. Alongside the specific elements of each intervention, a crucial factor for their effectiveness is the provision of encouragement, empathy, and emotional support.

### 4.1. Limitations

The cross-sectional designs of the baseline and follow-up studies preclude etiological inferences even though the overall design was longitudinal. Participants in the study were outpatients of a clinic of a single neurologist (A.S.P.) at a single academic hospital (Yale New Haven Hospital) which limits the generalizability of the findings to patients in similar healthcare settings. The study sample, both at baseline and follow-up, mainly included individuals who were older, white, male, married, and held a college degree, with baseline mild to moderate disabilities. The results might have differed with a more diverse sample. Potential unmeasured confounders, such as iatrogenic effects of medications used to treat PD, may have influenced the results (Fava & Rafanelli, 2019). Dyskinesia and motor symptoms were evaluated only at baseline through the MDS-UPDRS Part 3; participants may have developed worsening of PD symptoms at the time of follow-up. The study also assumed that all participants who had intact cognition at baseline maintained their cognitive status throughout the follow-up period. Retrospective recall was employed to assess three variables prior to the pandemic: perceived emotional–informational social support, family functioning, and loneliness. This recall may have been biased by the effects of the pandemic. DS and DS-II, used in the study, are psychometric tools based on the assumption that the underlying dimension of demoralization is continuous (Kissane et al., 2004; Robinson et al., 2016b). Using a clinimetric method, such as the Demoralization Scale of the Diagnostic Criteria for Psychosomatic Research Interview (DCPR-D), based on the assumption that demoralization is categorical (either exists or does not exist), might have offered a more in-depth understanding of the participants’ profiles (Fava et al., 2017). Conducting such interview, however, was practically impossible during the pandemic due to COVID-19 related restrictions. Loneliness was evaluated using an abbreviated three-item version of the R-UCLA scale, rather than the full version. Nonetheless, the abbreviated scale has shown adequate internal consistency, as well as convergent and discriminant validity (Hughes et al., 2004).

In conducting a sample size analysis at 80% power, *p* < 0.05 for 11 predictors with a medium effect size of 0.15 122 subjects would be needed and for a large effect size of 0.35, 59 subjects were required. For 7 predictors with an effect size of 0.15 and 0.25 103 and 49 subjects were needed, respectively. A larger sample size may have resulted in additional predictors being significant in the regression analyses.

### 4.2. Strengths

Participants were evaluated and diagnosed by a movement disorders neurologist at baseline. Scales widely recognized in research, including studies on PD, were used. In addition to standard assessments, variables not examined in previous research, such as loneliness experienced before the pandemic, subjective incompetence, and demoralization, were studied. The longitudinal design allowed the inclusion of variables that turned out to be significant, such as PDHRQoL before the pandemic. Variables related to the pandemic were explored, assessing factors that might increase patients’ vulnerability, such as inadequate perceived social support and prior traumatic experiences.

## 5. Conclusions

This follow-up study of PD patients during the COVID-19 pandemic extends the findings of previous studies on this topic by measuring variables not previously assessed, such as demoralization, subjective incompetence, family functioning, and emotional social support, and variables specifically related to the pandemic. Three significant variables associated with loneliness were identified, female sex, baseline PDHRQoL, and demoralization, with only female sex and demoralization correlating with loneliness specifically associated with the COVID-19 pandemic. These variables remained significant even after considering other variables such as depression and anxiety. The key finding of this study was that demoralization was the most important mental health factor associated with loneliness. This finding highlights the necessity for interventions aimed at restoring morale to be included as an essential component of any treatment plan designed to alleviate loneliness during public health emergencies that require social isolation, such as a pandemic. Effective interventions should also include the provision of emotional support to help individuals overcome loneliness and regain a sense of meaning, purpose, and coherence in their lives.

## Figures and Tables

**Table 1:** Overview of administered measures and instruments.

Measure	Baseline Instruments	COVID-19 Follow-Up Instruments
Loneliness		LON ^1^
Date of Study	Interview	Questionnaire
Gender	Medical record	
Birthdate	Medical record	Questionnaire
Education	Interview	
Race/ethnicity	Interview	
Marital status	Interview	Questionnaire
Household size	Interview	Questionnaire
Medical illness	Medical record	Questionnaire
General health perception		SF-36 subscale
Cognition	GPCOG	
PD Motor function	MDS-UPDRS-m	
PD Dyskinesia	Neurological exam	
PD Stage	Hoehn and Yahr	
PDHRQoL	PDQ-8	PDQ-8
Anxiety	GAD-7	GAD-7
Depression	PHQ-9	PHQ-9
Subjective incompetence	SIS	SIS
Demoralization	DS	DS-II
PTSD		PC-PTSD-5
Trauma history		BTQ
COVID-19 PTSD		PC-PTSD-5
Perceived stress	IES	
Social support	ISEL-SF	BS-6-SC ^1^
Resilience	BRS	BRS
Family functioning		BAFFS ^1^
Activities of daily living	Interview	
Cigarette smoking	Interview	Questionnaire
Alcohol use	Interview	Questionnaire
Cannabis use		Questionnaire
Impact of COVID-19		Questionnaire
COVID-19 Fear		FC-19S
COVID-19 Anxiety		CAS
COVID-19 Obsession		OCS
COVID-19 specific items		Questionnaire

^1^ Pre-COVID-19 was based on retrospective recall self-report during the follow-up study. Abbreviations: BAFFS: Brief Assessment of Family Functioning Scale; BRS: Brief Resilience Scale; BS-6-SC: Brief Social Support Scale; GAD-7: Generalized Anxiety Disorder-7; LON: 3-item version of the R-UCLA Loneliness Scale; PDHRQoL: Parkinson disease health-related quality of life; PDQ-8: Parkinson Disease Questionnaire-8; PHQ-9: Patient Health Questionnaire; SIS: Subjective Incompetence Scale.

**Table 2:** Cronbach alpha of measures and test–retest reliability.

Measure	Scale	Cronbach Alpha		Test–Retest	SD	SEM
		Baseline	COVID-19	Reliability		
Loneliness	LON	0.91 ^1^	0.88	0.82	1.13	0.48
PDHRQoL	PDQ-8	0.70	0.67	0.22	5.39	4.76
Anxiety	GAD-7	0.83	0.82	0.44	4.33	3.24
Depression	PHQ-9	0.79	0.81	0.50	4.16	2.94
Subjective incompetence	SIS	0.83	0.92	0.34	0.58	0.47
Demoralization ^2^	DS-II	0.86	0.92	0.41	1.06	0.81
Social support	BS-6-SC	0.93 ^1^	0.93	0.85	1.35	0.52
Resilience	BRS	0.89	0.88	0.51	0.85	0.60
Family functioning	BAFFS	081 ^1^	0.79	0.82	1.10	0.47
Perceived stress	IES	0.71				
General health perception	SF-36 subscale		0.75			
Social support	ISEL-SF	0.73				
Follow COVID-19 guidelines			0.58			
COVID-19 Fear	GC-19S		0.80			
COVID-19 Anxiety	CAS		0.80			
COVID-19 Obsession	OCS		0.77			

^1^ Pre-COVID-19 was based on retrospective recall self-report during the follow-up study. ^2^ DS at baseline 24-item scale limited to the 16 items in DS-II. Scores for DS and DS-II were converted to z-score for test–retest reliability. Abbreviations: BAFFS: Brief Assessment of Family Functioning Scale; BRS: Brief Resilience Scale; BS-6-SC: Brief Social Support Scale; CAS: Coronavirus Anxiety Scale; DS: Demoralization Scale; DS-II: Demoralization Scale-II; FC-19S: Fear of COVID-19 Scale; GAD-7: Generalized Anxiety Disorder-7; IES: Impact of Events Scale; ISEL-SF: Interpersonal Support Evaluation List-Short Form; LON: 3 item version of the R-UCLA Loneliness Scale; OCS: Obsession with COVID-19 Scale; PDHRQoL: Parkinson disease health-related quality of life; PDQ-8: Parkinson Disease Questionnaire-Short Form; PHQ-9: Patient Health Questionnaire; SF-36: 36 Item Short Form Survey; SIS: Subjective Incompetence Scale.

**Table 3:** Paired analysis of baseline or pre-COVID-19 and COVID-19 follow-up.

Variable	Scale	Baseline	Pre-COVID-19	Follow-Up	Statistical Test	*p*<
Loneliness ^1^	LON		1.6 ± 1.8	1.9 ± 1.8	Paired *t*-test	0.04
Emotional–informational social support ^1^	BS-6-SC		6.4 ± 2.3	6.2 ± 2.5	Paired *t*-test	0.13
Family functioning ^1^	BAFFS		4.8 ± 1.8	5.0 ± 1.9	Paired *t*-test	0.18
Alcohol use		41.8%		40.0%	McNemar	0.79
Tobacco use		3.9%		7.8%	McNemar	0.63
Married		74.5%		72.7%	McNemar	1.0
Household size		2.5 ± 1.1		1.3 ± 0.9	Paired *t*-test	0.001
Subjective Incompetence	SIS	0.3 ± 0.4		0.7 ± 0.6	Paired *t*-test	0.001
Demoralization ^2^	DS-II	0.01 ± 1.0		0.1 ± 0.9	Paired *t*-test	0.70
Resilience	BRS	4.1 ± 0.9		3.6 ± 0.8	Paired *t*-test	0.13
Anxiety (continuous)	GAD-7	4.0 ± 4.5		3.4 ± 3.5	Paired *t*-test	0.35
Anxiety (dichotomous)	GAD-7	9.6%		9.6%	McNemar	1.0
Depression (continuous)	PHQ-9	4.7 ± 4.2		5.1 ± 4.2	Paired *t*-test	0.59
Depression (dichotomous)	PHQ-9	11.1%		11.1%	McNemar	1.0
PDHRQoL	PDQ-8	4.9 ± 4.5		7.2 ± 4.1	Paired *t*-test	0.003

^1^ Pre-COVID-19 was based on retrospective recall self-report during the follow-up study. ^2^ DS at baseline 24-item scale limited to the 16 items in DS-II. Scores for DS and DS-II were converted to z-score for paired analysis. Abbreviations: BAFFS: Brief Assessment of Family Functioning Scale; BRS: Brief Resilience Scale; BS-6-SC: Brief Social Support Scale; DS: Demoralization Scale; DS-II: Demoralization Scale II; GAD-7: Generalized Anxiety Disorder-7; LON: 3-item version of the R-UCLA Loneliness Scale; PDHRQoL: Parkinson disease health-related quality of life; PDQ-8: Parkinson Disease Questionnaire-8; PHQ-9: Patient Health Questionnaire; SIS: Subjective Incompetence Scale.

**Table 4:** Bivariate analysis: association of continuous variables with loneliness during COVID-19 pandemic.

Variable	Scale	N	Mean ± SD	r	*p*<
Age		55	70.8 ± 8.8	−0.13	0.37
Loneliness, pre-COVID-19	LON	53	1.5 ± 1.8	0.81	0.001
Emotional–informational social support, follow-up	BS6-SC	54	6.1 ± 2.5	−0.33	0.02
Emotional–informational social support, pre-COVID-19	BS6-SC	52	6.5 ± 2.3	−0.34	0.02
Perceived social support, baseline	ISEL-SF	55	1.3 ± 0.5	−0.36	0.02
Family functioning, follow-up	BAFFS	55	5.0 ± 1.9	0.36	0.006
Family functioning, pre-COVID-19	BAFFS	53	4.8 ± 1.8	0.41	0.002
Household size, follow-up		55	1.3 ± 0.9	0.03	0.85
Household size, baseline		55	2.5 ± 1.1	0.26	0.06
Number of medical diagnoses, follow-up		55	0.4 ± 0.7	−0.03	0.83
General health perception, follow-up	SF36 Subscale	50	55.3 ± 20.9	−0.26	0.08
Resilience, follow-up	BRS	53	3.6 ± 0.83	−0.34	0.02
Resilience, baseline	BRS	55	4.1 ± 0.9	−0.41	0.002
Subjective incompetence, follow-up	SIS	54	0.7 ± 0.6	0.37	0.006
Subjective incompetence, baseline	SIS	55	0.3 ± 0.4	0.30	0.03
Demoralization, follow-up	DS-II	54	6.0 ± 5.9	0.52	0.001
Demoralization, baseline	DS	55	9.9 ± 11.4	0.36	0.007
Demoralization, follow-up (z-score)	DS-II	54	0.0 ± 1.0	0.52	0.001
Demoralization, baseline (z-score) ^1^	DS-II	52	0.0 ± 1.0	0.37	0.007
Depression follow-up continuous	PHQ-9	54	5.1 ± 4.2	0.46	0.001
Depression baseline continuous	PHQ-9	55	4.7 ± 4.2	0.35	0.01
Anxiety follow-up continuous	GAD-7	52	3.4 ± 3.5	0.41	0.003
Anxiety baseline continuous	GAD-7	55	3.9 ± 4.4	0.28	0.04
Pandemic impacted life		55	2.5 ± 0.94	0.05	0.72
Fear of COVID-19	FC-19S	54	18.6 ± 5.7	0.21	0.13
Coronavirus Anxiety	CAS	54	0.6 ± 1.4	0.23	0.10
Obsession with COVID-19	OCS	54	1.0 ± 1.8	0.12	0.40
Quality of life, follow-up	PDQ-8	50	7.2 ± 4.1	0.38	0.007
Quality of life, baseline	PDQ-8	55	4.9 ± 4.4	0.46	0.001
Perceived Stress, baseline	IES	55	11.0 ± 9.3	0.07	0.19
Follow COVID-19 guidelines		54	32.5 ± 4.3	−0.05	0.74
Years since diagnosis, baseline		55	3.0 ± 1.1	−0.04	0.79
Cognition, baseline	GPCOG	42	8.3 1.3	0.03	0.87
Motor symptoms, baseline	MDS-UPDRS-m	55	23.56 ± 10.6	0.21	0.12
Hoehn and Yahr stage, baseline	H-Y	55	0.20 ± 0.40	0.16	0.25

^1^ DS at baseline 24-item scale limited to the 16 items in DS-II. Scores for DS and DS-II were converted to z-score. Abbreviations: BAFFS: Brief Assessment of Family Functioning Scale; BS-6-SC: Brief Social Support Scale; BRS: Brief Resilience Scale; CAS: Coronavirus Anxiety Scale; DS: Demoralization Scale; DS-II: Demoralization Scale-II; FC-19S: Fear of COVID-19 Scale; GPCOG: General Practitioner Assessment of Cognition Screening Test; H-Y: Hoehn and Yahr staging system; IES: Impact of Event Scale; ISEL-SF: Interpersonal Support Evaluation List; LON: 3-item version of the R-UCLA Loneliness Scale; MDS-UPDRS-m: Part III of the Movement Disorder Society Sponsored Revision of the Unified Parkinson Disease Rating Scale for motor symptoms; OCS: Obsession with COVID-19 Scale; PDQ-8: Parkinson Disease Questionnaire-8; Short Form; SF-36: 36-item Short Form Survey; SIS: Subjective Incompetence Scale.

**Table 5:** Bivariate analysis: Association of categorical variables with loneliness during the COVID-19 pandemic.

Variable		N	Mean ± SD	t	df	*p*<
Sex	Male	34	1.3 ± 1.5	3.5	53	0.001
	Female	21	2.9 ± 1.9			
Married, follow-up	Yes	40	1.4 ± 1.5	3.3	53	0.002
	No	15	3.1 ± 2.1			
College Education	Yes	42	1.9 ± 2.0	−0.1	53	0.89
	No	13	1.8 ± 1.5			
Had COVID-19	Yes	2	4.5 ± 2.1	−2.3	51	0.03
	No	51	1.7 ± 1.7			
Tested for COVID-19	Yes	23	2.0 ± 2.0	−0.3	53	0.76
	No	32	1.8 ± 1.8			
Family member had	Yes	18	1.6 ± 1.6	0.8	53	0.41
COVID-19	No	37	2.1 ± 1.9			
Alcohol use, follow-up	Yes	22	1.7 ± 1.6	0.4	53	0.66
	No	33	2.0 ± 2.0			
Tobacco use, follow-up	Yes	4	2.0 ± 1.9	−0.1	53	0.92
	No	51	1.9 ± 1.8			
Cannabis use, follow-up	Yes	5	0.6 ± 1.3	1.6	50	0.11
	No	47	2.0 ± 1.9			
Depression, follow-up	Yes	6	3.8 ± 1.7	−3.1	52	0.003
(PHQ-9)	No	48	1.6 ± 1.7			
Anxiety, follow-up	Yes	5	3.4 ± 1.5	−2.0	50	0.06
(GAD-7)	No	47	1.7 ± 1.8			
Depression, baseline	Yes	6	3.0 ± 1.8	−1.6	53	0.06
(PHQ-9)	No	49	1.8 ± 1.8			
Anxiety, baseline	Yes	5	3.2 ± 1.9	−1.7	53	0.1
(GAD-7)	No	50	1.8 ± 1.8			
Dyskinesia, baseline	Yes	11	2.2 ± 1.7	−0.5	53	0.59
	No	44	1.8 ± 1.9			

Abbreviations: PHQ-9: Patient Health Questionnaire-9; GAD-7: Generalized Anxiety Disorder-7; MDS-UPDRS-m: Part III of the Movement Disorder Society Sponsored Revision of the Unified Parkinson Disease Rating Scale for motor symptoms.

**Table 6:** Linear regression models with loneliness during COVID-19 pandemic as the dependent variable.

Model 1: Independent variables during COVID-19 pandemic associated with COVID-19 loneliness							
**R**	**R^2^**	**Adj R^2^**	**SEE**	**R^2^ change**	**F change**	**df1**	**df2**
0.639	0.408	0.372	1.477	0.408	11.267	3	49
***p*** **<**	**AIC**	**APC**	**MPC**	**SBC**			
0.001	45.151	0.688	4.000	53.032			
			**SS**	**df**	**MS**	**F**	***p*** **<**
Regression			73.696	3	24.565	11,267	0.001
Residual			106.832	49	2.180		
Total			180.528	52			
			**b**	**SE**	**β**	**t**	***p*** **<**
(Constant)			4.428	1.085		4.083	0.001
Demoralization, follow-up			0.104	0.039	0.329	2.649	0.011
Sex			−1.234	0.459	−0.324	−2.689	0.010
Emotional–informational social support, follow-up			−0.187	0.083	−0.257	−2.258	0.028
			**Tolerance**	**VIF**			
Demoralization, follow-up			0.78	1.28			
Sex			0.83	1.20			
Emotional–informational social support, follow-up			0.94	1.07			
Excluded variables: Depression, family functioning, had COVID-19, marital status, PDHRQoL, resilience, sex, and subjective incompetence during COVID-19 follow-up							
Model 2: Independent variables at baseline associated with COVID-19 loneliness							
**R**	**R^2^**	**Adj R^2^**	**SEE**	**R^2^ change**	**F change**	**df1**	**df2**
0.461	0.212	0.197	1.647	0.212	14.287	1	53
***p*** **<**	**AIC**	**APC**	**MPC**	**SBC**			
0.001	56.854	0.847	2.000	60.869			
			**SS**	**df**	**MS**	**F**	***p*** **<**
Regression			38.760	1	38.760	14.287	0.001
Residual			143.785	53	2.713		
Total			182.545	54			
			**b**	**SE**	**β**	**t**	***p*** **<**
(Constant)			0.971	0.333		2.916	0.005
PDHRQoL, baseline			0.192	0.051	0.461	3.780	0.001
Excluded variables: Family functioning prior to COVID-19, emotional–informational social support prior to COVID-19, demoralization, social support, and subjective incompetence at baseline							
Model 3: Best explanatory model of risk factors for loneliness during COVID-19 pandemic							
**R**	**R^2^**	**Adj R^2^**	**SEE**	**R^2^ change**	**F change**	**df1**	**df2**
0.710	0.503	0.682	1.367	0.503	12.168	4	48
***p*** **<**	**AIC**	**APC**	**MPC**	**SBC**			
0.001	37.850	0.600	5.000	47.702			
			**SS**	**df**	**MS**	**F**	***p*** **<**
Regression			90.891	4	22.723	12.168	0.001
Residual			89.637	48	1.867		
Total			180.528	52			
			**b**	**SE**	**β**	**t**	***p*** **<**
(Constant)			3.502	1.049		3.337	0.002
PDHRQoL, baseline			0.135	0.044	0.321	3.034	0.004
Demoralization, follow-up			0.087	0.037	0.277	2.387	0.021
Sex			−1.151	0.426	−0.302	−2.704	0.009
Emotional–informational social support, follow-up			−0.151	0.078	−0.207	−1.949	0.057
			**Tolerance**	**VIF**			
PDHRQoL, baseline			0.93	1.08			
Demoralization, follow-up			0.77	1.30			
Sex			0.83	1.21			
Emotional–informational social support, follow-up			0.91	1.09			
Model 4: Risk factors for loneliness specific to COVID-19 pandemic controlled for pre-COVID loneliness							
**R**	**R^2^**	**Adj R^2^**	**SEE**	**R^2^ change**	**F change**	**df1**	**df2**
0.870	0.757	0.730	0.974	0.757	28.013	5	45
***p*** **<**	**AIC**	**APC**	**MPC**	**SBC**			
0.001	2.964	0.308	6.000	14.555			
			**SS**	**df**	**MS**	**F**	***p*** **<**
Regression			132.967	5	26.593	28.013	0.001
Residual			42.720	45	0.949		
Total			175.686	50			
			**b**	**SE**	**β**	**t**	***p*** **<**
(Constant)			2.000	0.899		2.466	0.018
Loneliness, prior to COVID-19 pandemic			0.629	0.097	0.620	6.461	0.001
PDHRQoL, baseline			0.026	0.037	0.062	0.691	0.493
Demoralization, follow-up			0.066	0.029	0.199	2.299	0.026
Sex			−0.737	0.396	−0.192	−2.334	0.004
Emotional–informational social support, follow-up			0.026	0.037	0.062	0.691	0.493
			**Tolerance**	**VIF**			
Loneliness, prior to COVID-19 pandemic			0.59	1.25			
PDHRQoL, baseline			0.68	1.47			
Demoralization, follow-up			0.72	1.39			
Sex			0.80	1.25			
Emotional–informational social support, follow-up			0.86	1.16			

Abbreviations: R^2^ = R Square; Adj R^2^ = Adjusted R Square; SSE = Standard Error of the Estimate; AIC = Akaike Information Criterion; APC = Amemiya Prediction Criterion; MPC = Mallows’ Prediction Criterion; SBC = Schwarz Bayesian Criterion; Tolerance = Tolerance test of Multicollinearity; VIF = Variance Inflation Factor for Multicollinearity.

## Data Availability

The data presented in this study are available in Yale Dataverse: https://doi.org/10.60600/YU/FCGFJS.

## Abbreviations

The following abbreviations are used in this manuscript:

Adj R^2^	Adjusted R Square
AIC	Akaike Information Criterion
APC	Amemiya Prediction Criterion
BAFFS	Brief Assessment of Family Functioning Scale
BRS	Brief Resilience Scale
BS-6-SC	Brief Social Support Scale
BTQ	Brief Trauma Questionnaire
CAS	Coronavirus Anxiety Scale
DS	Demoralization Scale
DS-II	DS-II: Demoralization Scale-II
FC-19S	Fear of COVID-19 Scale
GAD-7	Generalized Anxiety Disorder-7
GPCOG	General Practitioner Assessment of Cognition Screening Test
HRQoL	Health-related quality of life
H-Y	Hoehn and Yahr staging system
IES	Impact of Event Scale
ISEL-SF	Interpersonal Support Evaluation List Short Form
LON	LON: 3-item version of the R-UCLA Loneliness Scale
MDS-UPDRS-m	Part III of the Movement Disorder Society Sponsored Revision of the Unified Parkinson Disease Rating Scale for motor symptoms
MPC	Mallows’ Prediction Criterion
OCS	Obsession with COVID-19 Scale
PD	Parkinson disease
PDHRQoL	Parkinson disease health-related quality of life
PDQ-8	Parkinson Disease Questionnaire-8
PHQ-9	Patient Health Questionnaire
PTSD	Post-traumatic stress disorder
PC-PTSD-5	Primary Care PTSD-5 Scale
R^2^	R Square
SBC	Schwarz Bayesian Criterion
SF-36	36-item Short Form Survey
SIS	Subjective Incompetence Scale
SSE	Standard Error of the Estimate
